# Implications of polygenic risk-stratified screening for prostate cancer on overdiagnosis

**DOI:** 10.1038/gim.2014.192

**Published:** 2015-01-08

**Authors:** Nora Pashayan, Stephen W. Duffy, David E. Neal, Freddie C. Hamdy, Jenny L. Donovan, Richard M. Martin, Patricia Harrington, Sara Benlloch, Ali Amin Al Olama, Mitul Shah, Zsofia Kote-Jarai, Douglas F. Easton, Rosalind Eeles, Paul D. Pharoah

**Affiliations:** 1 Department of Applied Health Research, University College London, London, UK; 2 Centre for Cancer Prevention, Mathematics and Statistics, Wolfson Institute of Preventive Medicine, Queen Mary University of London, London, UK; 3 Cancer Research UK Cambridge Institute, University of Cambridge, Cambridge, UK; 4 Nuffield Department of Surgery, Oxford John Radcliffe Hospital, University of Oxford, Headington, Oxford, UK; 5 School of Social and Community Medicine, University of Bristol, Bristol, UK; 6 Centre for Cancer Genetic Epidemiology, Department of Public Health and Primary Care, Strangeways Research Laboratory, University of Cambridge, Cambridge, UK; 7 Department of Oncology, Strangeways Research Laboratory, University of Cambridge, Cambridge, UK; 8 Division of Genetics and Epidemiology, Institute of Cancer Research & Royal Marsden NHS Foundation Trust, London, UK

**Keywords:** overdiagnosis, polygenic risk, prostate cancer, risk-stratified screening

## Abstract

**Purpose:**

This study aimed to quantify the probability of overdiagnosis of prostate cancer by polygenic risk.

*Genet Med* **17** 10, 789–795.

**Methods:**

We calculated the polygenic risk score based on 66 known prostate cancer susceptibility variants for 17,012 men aged 50–69 years (9,404 men identified with prostate cancer and 7,608 with no cancer) derived from three UK-based ongoing studies. We derived the probabilities of overdiagnosis by quartiles of polygenic risk considering that the observed prevalence of screen-detected prostate cancer is a combination of underlying incidence, mean sojourn time (MST), test sensitivity, and overdiagnosis.

*Genet Med* **17** 10, 789–795.

**Results:**

Polygenic risk quartiles 1 to 4 comprised 9, 18, 25, and 48% of the cases, respectively. For a prostate-specific antigen test sensitivity of 80% and MST of 9 years, 43, 30, 25, and 19% of the prevalent screen-detected cancers in quartiles 1 to 4, respectively, were likely to be overdiagnosed cancers. Overdiagnosis decreased with increasing polygenic risk, with 56% decrease between the lowest and the highest polygenic risk quartiles.

*Genet Med* **17** 10, 789–795.

**Conclusion:**

Targeting screening to men at higher polygenic risk could reduce the problem of overdiagnosis and lead to a better benefit-to-harm balance in screening for prostate cancer.

*Genet Med* **17** 10, 789–795.

## Introduction

The recently updated US Preventive Services Task Force guidelines recommended against serum prostate-specific antigen (PSA)-based screening for prostate cancer on the grounds that the expected harms of screening (false-positive findings, overdiagnosis, and overtreatment) outweigh the potential benefits.^1^ Conceptually, overdiagnosis is defined as detection by screening of tumors that would not have presented clinically in a person's lifetime in the absence of screening.

The US Preventive Services Task Force recommendation was based primarily on evidence from the two largest multicenter randomized screening trials. The US Prostate, Lung, Colorectal, and Ovarian Cancer Screening Trial involved 76,000 men aged 55 to 74 years who were randomized to either the screening arm and offered PSA test annually for 6 years or the control arm and received usual care. After 13 years of follow-up, the US Prostate, Lung, Colorectal, and Ovarian Cancer Screening Trial found no difference in prostate cancer-specific mortality between the two arms of the trial. However, more than 40% of men in the control arm underwent PSA testing. This study shows that there is no prostate cancer–related mortality benefit of organized annual screening compared with opportunistic screening.^2^ By contrast, the European Randomized Study of Screening for Prostate Cancer involved 182,000 men aged 50 to 74 years randomized to the screening arm and offered PSA testing, on average, every 4 years, or to the control arm with no intervention offered. The European Randomized Study of Screening for Prostate Cancer showed a 21% reduction in prostate cancer–related mortality after a median follow-up of 13 years. To prevent one death from prostate cancer, 781 men would need to be invited for screening and 27 additional cancers would need to be detected.^3^ These findings indicate that population screening for prostate cancer using PSA can prevent death from the cancer for a subset of men, but at a cost of overdiagnosis and subsequent overtreatment. This raises the question of whether targeted screening for prostate cancer can improve the benefit-to-harm ratio of screening.

Advances in genomics raise expectations of moving from the conventional “one-size-fits-all” to more personalized or risk-stratified screening approach. By stratifying the population into several groups according to genetic risk alone or combined with other risk factors (such as age and family history), screening could be offered differentially to each population stratum with potentially improved benefit-to-harm ratio.^4,5^

To date, genome-wide association studies have identified 76 prostate cancer susceptibility loci, to which ~30% of the familial risk of prostate cancer can be attributed.^6^ Assuming a log-additive model of interaction between loci, these loci define a polygenic risk profile with a variance for the log relative risk distribution of 0.43 and estimated relative risks at the 5th and 95th percentiles of 0.27 and 2.36, respectively.^7^ Such a distribution is limited in predicting prostate cancer for any given individual because discrimination is limited (the area under the receiver operator characteristic curve is 0.68) (ref. ^8^); however, it could be used for risk stratification in prevention programs at the population level.^6^

A risk-stratified screening strategy for prostate cancer with eligibility for screening based on an absolute risk that is dependent on age and polygenic risk profile has been shown to have the potential to improve the efficiency of the screening program by reducing the number of men invited to undergo screening while detecting most cancers potentially detectable by a conventional age-based screening strategy.^5^ However, there is no evidence from empirical data that such personalized screening strategy would reduce harms of screening by reducing overdiagnosis and subsequent overtreatment. Using mathematical modeling, this study aims at estimating the proportion of screen-detected cancers likely to be overdiagnosed by quartiles of polygenic risk profile.

## Materials and Methods

### Study participants

We obtained genotyping and clinicopathological data for 17,012 men aged 50 to 69 years (9,404 men identified with prostate cancer and 7,608 with no cancer) from three UK-based studies: Prostate Testing for Cancer and Treatment (ProtecT),^9,10^ Studies of Epidemiology and Risk Factors in Cancer Heredity (SEARCH), and UK Genetic Prostate Cancer Study (UKGPCS)^11^ (**Supplementary Table S1** online). The ProtecT study provided prevalence screen data, and SEARCH and UKGPCS provided the clinical incidence data. Details of each of the studies have been published previously.

The ProtecT study is a UK-wide study of community-based PSA testing and randomized trial of subsequent prostate cancer treatment.^9^ PSA testing in the context of the ProtecT study is akin to prevalence screening. Between 2001 and 2008, ~200,000 men between the ages of 50 and 69 years, ascertained through randomly selected general practices in nine regions in the United Kingdom, were invited for enrollment. Men who consented and met the eligibility criteria (82,429 men) were offered a PSA test. All men with PSA ≥3.0 ng/ml were offered transrectal ultrasound-guided biopsy using a 10-core lateral biopsy template. DNA extracts were available for 2,148 (74%) cases and for 6,648 men with no prostate cancer who provided consent for their blood samples to be used for genetic studies. Given that 2,895 men of 82,429 men were diagnosed with prostate cancer as a result of PSA testing, then 2,148 cases would have been detected from a population of 61,160 PSA-tested men.

SEARCH is a population-based case–control study. Eligible cases are men younger than 70 years of age and registered with prostate cancer at the Eastern Cancer Registration and Information Centre (ECRIC) population-based cancer registry in East of England. Controls are men attending general practice who are frequency matched to cases by age and geographic region (http://ccge.medschl.cam.ac.uk/search-study/). Genotyping data were available for all SEARCH cases (*n* = 4,099) and controls (*n* = 960) aged 50–69 years.

The UKGPCS^11^ includes men from families with multiple cases of prostate cancer, men diagnosed with prostate cancer at 60 years of age or younger throughout the United Kingdom, and systematic series from the prostate cancer clinic at the Royal Marsden NHS Foundation Trust. Genotyping data were available for 3,157 cases.

Prostate cancer was classified as localized disease with tumor-node-metastasis stage T2 and below and advanced disease (regional-distant) was classified as localized disease with tumor-node-metastasis stage T3 and above (low aggressive tumor, Gleason score <7; intermediate to highly aggressive tumor, Gleason score ≥7).

### Genotyping and quality control

We obtained the genotyping data in two rounds. Genotyping outcome for 11,737 men on 70 known prostate cancer susceptibility single-nucleotide polymorphisms (SNPs) were obtained from genotyping performed using a custom Illumina Infinium array (iCOGS), as described previously.^7^ ProtecT and SEARCH participants (*N* = 5,275) not genotyped using the iCOGS array were genotyped for 60 of these SNPs and for 10 highly correlated surrogate SNPs (*r*^2^ > 0.75) using Fluidigm BioMark 96.96 Dynamic Array (Fluidigm, South San Francisco, CA) and TaqMan SNP assays (BioTrove, Woburn, MA) according to the manufacturer's instructions.^12^ Both genotyping rounds were performed in the same laboratory. In each 384-well plate, 2% duplicates and 1% PCR-negative controls (with no DNA) were included. Genotype intensity cluster plots were visually inspected, and the data were excluded if clustering was judged to be poor. We excluded individuals with more than 10% missing genotypes and excluded variants without genotype call (rs3850699, rs6062509, rs7210100), variants with a call rate less than 90% (rs675495), and variants that deviated from Hardy–Weinberg equilibrium in controls at *P* < 0.005 (rs1465618). To harmonize the genotype calls in the two rounds of genotyping, when a variant was excluded, its surrogate marker was also excluded (rs339331 as surrogate SNP to rs675495).

Hence, the analysis was based on data from 66 loci associated with genome-wide significance level with susceptibility for prostate cancer in European populations (**Supplementary Table S2** online).

### Polygenic risk score and absolute risk calculations

For individuals with missing genotype, we imputed the expected value based on the observed allele frequency. A polygenic risk score (PRS) for each individual was calculated as:

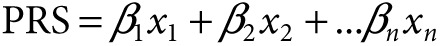


where βn is the per-allele log odds ratio for variant n (Supplementary **Table S2** online), and Xn represents the number of risk alleles carried by each individual at locus n (i.e., 0, 1, or 2). Thus, the risk conferred by each of the variants is assumed to be allele dose dependent with a multiplicative (log-additive) effect on a relative risk scale.^13^ Under the multiplicative model, the distribution of polygenic risk in the population at birth follows the normal distribution when relative risk is plotted on a logarithmic scale, with mean, *μ*, and variance *σ*^2^. We set the mean, *μ* = −*σ*^2^/2, so that the mean relative risk in the population at birth is equal to unity. The distribution of relative risk among cases at young ages is also log-normal with the same variance, but with a larger mean, *μ* + *σ*^2^ (ref. 13). Details are described in the **Supplementary Data** online.

We calculated the age-specific absolute risk for developing prostate cancer as:

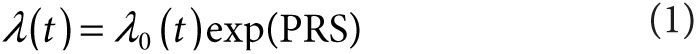


where 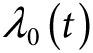 is the baseline absolute risk in the population at age t, derived from the number of prostate cancer registrations, deaths from the cancer, deaths from all causes, and mid-year population estimates for England from 2002 to 2006, using DevCan 6.4.1 software (http://surveillance.cancer.gov/devcan/).

### Derivation of estimates of probabilities of overdiagnosis

The observed prevalence of screen-detected prostate cancer is a combination of the underlying incidence, the duration of the preclinical detectable period (mean sojourn time (MST)), test sensitivity, and overdiagnosis.

The observed prevalence in polygenic risk stratum *j* is

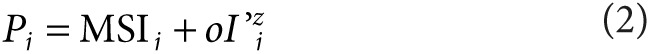


where *M* represents MST, *S* represents test sensitivity, and *I*_*j*_ represents the underlying age-conditional 1-year absolute risk for developing cancer in polygenic risk stratum *j* (all pertaining to the nonoverdiagnosed cancers), 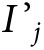
represents the age-conditional 1-year absolute risk among the clinically detected cases, and 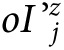 is the rate of overdiagnosis in stratum *j*. In this model, we posit that the logarithm of the rate of overdiagnosis is a linear function of the rate of clinically detected cases. In this model, *o* is the exponential of the constant term and *z* is the slope of the relationship in the logarithmic scale between incidence of clinical disease and overdiagnosed cancer.

We divided the polygenic risk distribution among the population into quartiles. We calculated the observed prevalence of prostate cancer by risk quartiles within the ProtecT trial from the number of ProtecT cases and the extrapolated number of men tested in the ProtecT study within each risk quartile. We derived the incidence of clinically detected cancer by risk quartiles from SEARCH and UKGPCS after excluding cases recorded as screen detected. Assuming sojourn time and test sensitivity of nonoverdiagnosed cancers to be equal across the risk quartiles, we estimated the unknown parameters, *o* and *z*, from equation (2) across the four strata, such that:

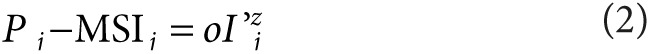


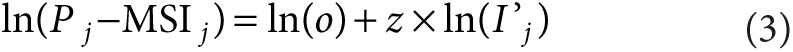


In a sensitivity analysis, we varied the sojourn time and the test sensitivity and derived estimates of the unknown parameters.

## Results

The distribution of the PRS based on 66 prostate cancer susceptibility variants had mean of −0.004 and variance of 0.40 among men 50 to 69 years with no prostate cancer, and mean of 0.39 and variance of 0.40 among men with the cancer. The PRSs at the 25th, 50th, and 75th percentiles of the scaled risk distribution were −0.63, −0.20, and 0.23, respectively (**Table 1**). Polygenic risk quartiles 1 to 4 accounted for 9, 18, 25, and 48% of the cases, respectively (**Table 2**).

There was no statistically significant association between quartiles of PRS and stage (χ^2^ = 1.72; *P* = 0.63) or Gleason score categories (χ^2^ = 0.64; *P* = 0.93; **Table 3**). PSA level was available for 6,410 men with prostate cancer and for 6,646 men without prostate cancer. PSA level varied by polygenic risk, 33% of men in polygenic risk quartile 1 compared with 66% of men in quartile 4 had PSA ≥4 ng/ml (χ^2^ = 791.1; *P* < 0.001). After adjusting for stage and Gleason score, with every 1-point increase in PRS, the odds of having PSA ≥4 ng/ml compared with PSA < 4 ng/ml increased 1.23-fold (95% confidence interval: 1.10–1.37; *P* < 0.001).

**Table 4** presents the observed prevalence of screen-detected cancer and incidence of clinically detected cancer by polygenic risk quartile. Assuming MST for nonoverdiagnosed cancers of 9 years^14^ and test sensitivity of 80%,^14^ the estimated *o* was 0.07 and *z* was 0.37. A value of *z* of <1 indicates that the increase in incidence of clinical disease by risk score is greater than the increase in overdiagnosed disease. The rates of overdiagnosed cases estimated were 6, 8, 9, and 12 per 1,000 in quartiles 1–4, respectively. However, the prevalence of all cancers increased more rapidly with risk quartile at 15, 26, 36, and 63 per 1,000. Thus, although the absolute rate of overdiagnosis was estimated to increase with polygenic risk, the proportion of screen-detected cancers that are likely to be overdiagnosed decreased with an increase in polygenic risk quartiles. Therefore, 43, 30, 25, and 19% of the cases were likely to be overdiagnosed in polygenic risk quartiles 1 to 4, respectively. The proportion of overdiagnosed cases was 56% lower in highest polygenic risk quartile compared with the lowest quartile.

**Table 5** presents the proportion of the screen-detected cancers likely to be overdiagnosed by quartiles of polygenic risk in a sensitivity analysis using MST between 8 and 10 years and PSA test sensitivity between 80 and 90% and varying these parameters with risk quartiles, assuming shorter MST and higher test sensitivity with higher polygenic risk quartiles. The absolute value of the proportion of cancers overdiagnosed changed with changing these parameters; however, the pattern of lower proportion of cancers likely overdiagnosed with increasing polygenic risk quartile remained.

## Discussion

To our knowledge, this is the first study to show that the proportion of overdiagnosed cases in prostate cancer screening is likely to vary inversely by polygenic risk. The findings suggest that a polygenic risk-stratified screening strategy could reduce the problem of overdiagnosis.

Targeting screening to men at higher polygenic risk would reduce number of men likely to be overdiagnosed at a cost of detecting fewer men with cancer. Reducing the number of men invited to screening would further reduce harms associated with screening, such as false-positive findings and their diagnostic work-up. The optimal risk threshold will be population- and health care–specific. Preferences of men, organization of the screening program, the resource implications, economic evaluation, and assessment of the potential benefits and harms of a stratified screening program would all need to be explored to inform the optimal risk threshold.

It is likely that the trade-off between reducing overdiagnosed cancers and missing progressive cancers would further improve as more susceptibility loci are identified^5^ and as additional information is incorporated into the risk score, such as family history^15,16^ and baseline PSA level.^17^ Further studies are needed using empirical data to test the implications of adding information regarding baseline PSA test level and family history to polygenic risk profiling for personalized screening in prostate cancer.

The individual and cumulative associations of the susceptibility loci with prostate cancer aggressiveness remain inconclusive.^18,19^ In our study, there was no association between PRS and stage or Gleason score. The absence of association between Gleason score and polygenic risk is consistent with our fundamental assumption of a common MST by PRS. The proportion likely overdiagnosed was found to be inversely associated with polygenic risk. It may be that genetic profile is associated with progression of disease independent of stage and Gleason score. Overdiagnosis depends not only on tumor biology but also on the life expectancy of the individual. An aggressive tumor still could be considered overdiagnosed if a person dies before the tumor presents symptomatically. To account for competing causes and age, we have used age-conditional absolute risk for developing prostate cancer in deriving the expected prevalence by polygenic risk quartiles.

Our data on screen-detected cancers come from prevalence screening. Consequently, due to the absence of data regarding interval cancers and incident screen-detected cancer, we could not derive the MST and PSA test sensitivity for different polygenic risk strata using multi-state modeling. Previously, we derived the MST from prevalence data derived from the ProtecT study and the incidence data derived from English cancer registration among men 50 to 69 years, taking into account the sensitivity of the PSA test estimated from external data.^14^ We have estimated PSA test sensitivity as 80% and MST as 11 years.^14^ Our estimate of PSA test sensitivity is comparable to the estimate of 85% determined by Hakama et al.^20^ using the incidence method based on randomized prostate cancer screening trial in Finland. The incidence method is free from overdiagnosis bias.^20^ As such, this estimate is appropriate to use to derive the prevalence of progressive cancers. Our estimate of MST is comparable to the estimates from the European Randomized Study of Screening for Prostate Cancer. In the European Randomized Study of Screening for Prostate Cancer, the mean lead time ranges between 4 and 8 years.^21^ The mean lead time is approximately half the duration of the sojourn time for prevalence screening.^22^ Our estimate of the MST is based on prevalent cases, which include both progressive and overdiagnosed cancers. As such, it is expected that the sojourn time of progressive disease will be shorter than 11 years.

It is not known if the test sensitivity and tumor behavior vary by genetic risk. It may be that the test sensitivity is higher in detecting cancers with higher genetic risk and these cancers have shorter sojourn time. In a sensitivity analysis, we have used values for MST for progressive cancer between 8 and 10 years and for PSA test sensitivity between 80 and 90%. Previously using the same prevalent screen data, ProtecT data, we have estimated that approximately one-quarter of the screen-detected cases among men aged 50–69 years would be overdiagnosed.^14^ Our weighted average estimates of probabilities of overdiagnosis across the risk strata gave comparable figures when taking sojourn time of 9 years with 80% test sensitivity or varying sojourn time and test sensitivity across risk strata as 8 and 10 years and 80 and 90% for polygenic risk quartiles 1 and 2 and quartiles 3 and 4, respectively.

Evidence from randomized screening trial on probabilities of overdiagnosis, natural history of the disease, screening test performance, and cancer-specific mortality stratified by risk is warranted. This is to estimate the absolute benefits and harms of screening by polygenic risk threshold. Further research is needed to explore whether genetic testing for stratification and eligibility for screening would be acceptable to health professionals and to the public, and how the public will perceive not offering or less frequent offering of screening to subgroups of the population at low risk for cancer.^23^

In summary, tailoring of screening strategies to different risk groups may improve the balance between benefits and harms of screening. Targeting screening to men at higher polygenic risk could potentially reduce the problem of overdiagnosis in prostate cancer.

## Disclosure

R.E. declares receiving educational grants from GenProbe, Vista Diagnostics, and Illumina, an honorarium and expenses for the 2012 UK Cancer Convention from Succint Communications, and medical education support from Janssen for the 2013 Genitourinary Cancers Symposium of the American Society of Clinical Oncology. The other authors declare no conflict of interest.

## Figures and Tables

**Table 1:**
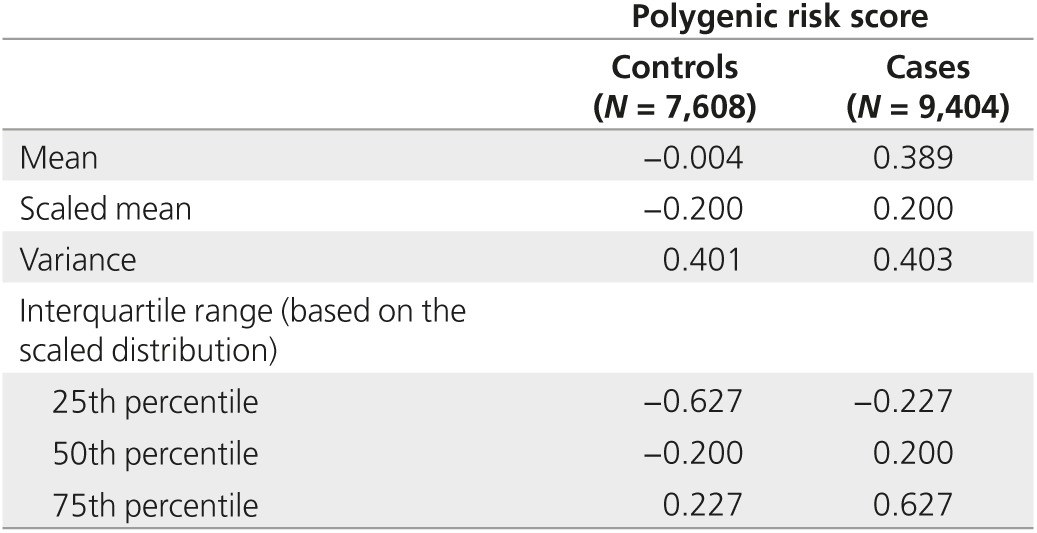
Polygenic risk distribution among men 50–69 years old with and without cancer in ProtecT, SEARCH, and UKGPCS studies based on 66 prostate cancer susceptibility loci

**Table 2:**
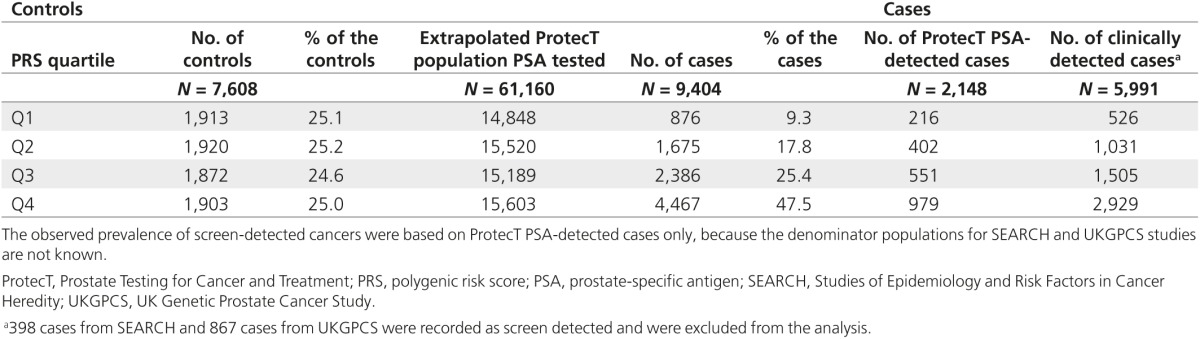
Number of men 50–69 years old with and without cancer, by quartile of polygenic risk

**Table 3:**
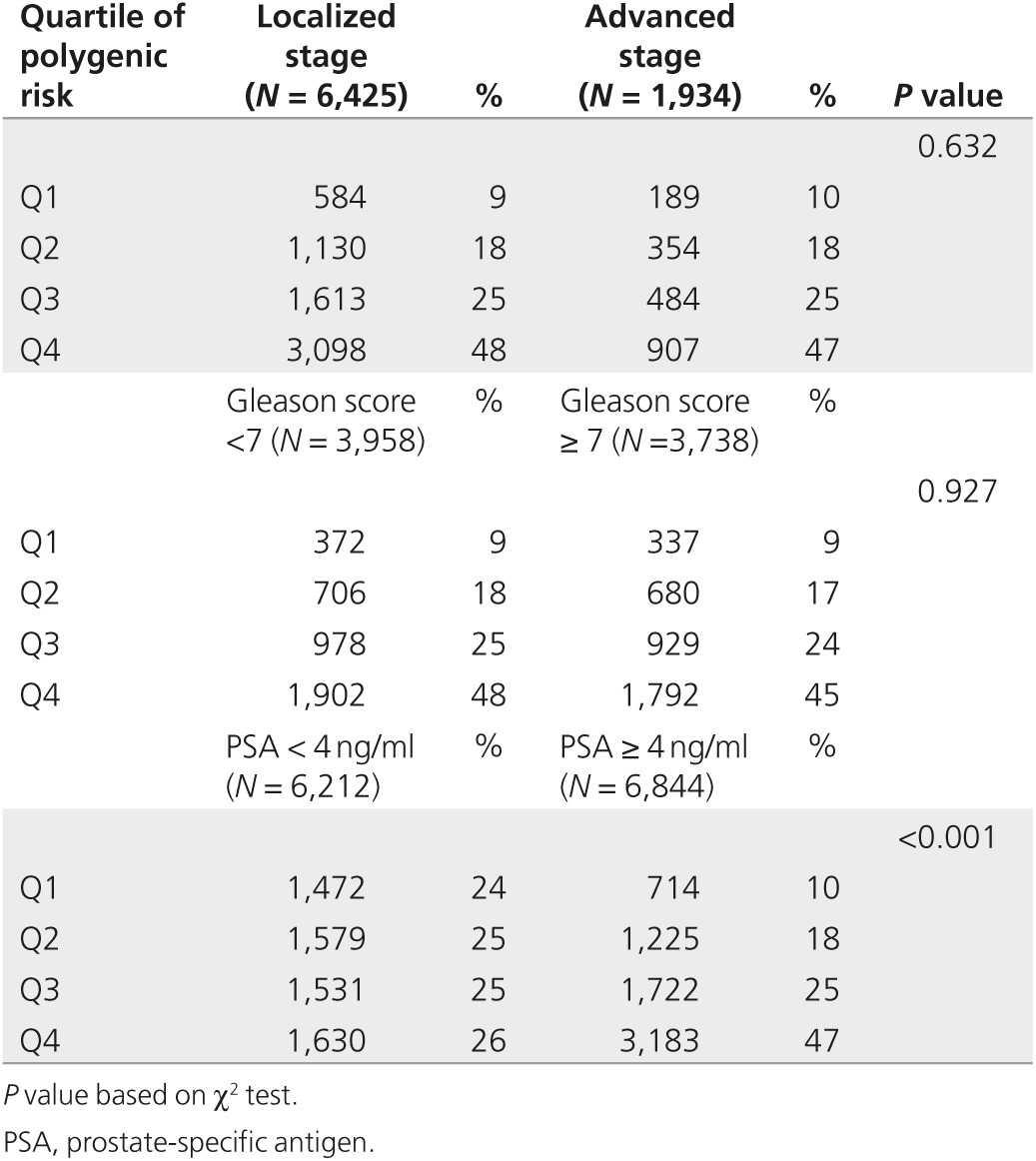
Association of stage, Gleason score, and PSA with quartile of polygenic risk

**Table 4:**
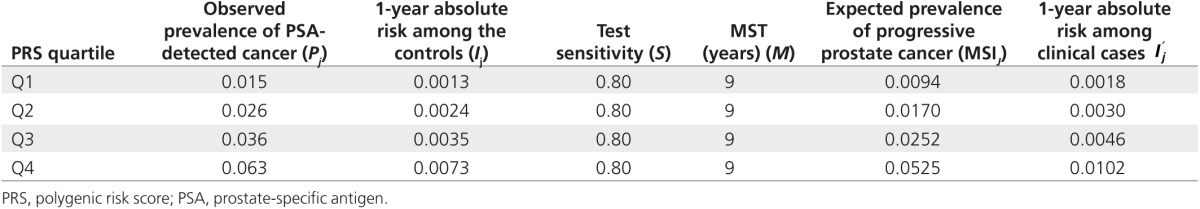
Summary of the inputs in estimating rate of overdiagnosis, by polygenic risk quartile

**Table 5:**
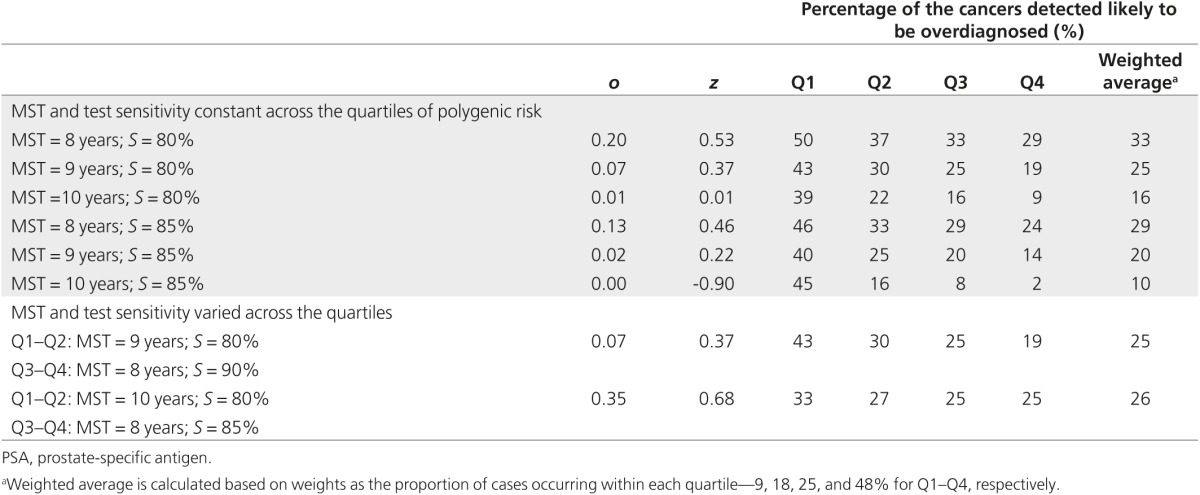
Sensitivity analysis with varying mean sojourn time (MST) and PSA test sensitivity (*S*) and the proportion of cases likely overdiagnosed, by quartile of polygenic risk
