# Supplementary material for: Implications of polygenic risk-stratified screening for prostate cancer on overdiagnosis
Source: Genet Med. 2015 Jan 8;17(10):789–95. doi: 10.1038/gim.2014.192 (PMC4430305; doi:10.1038/gim.2014.192)
Supplement: Supplementary Information [file gim2014192x1.doc]

**Description of additional data files**

File name: Supplementary file.doc

Title: Log-normal Risk Distribution

File name: Supplementary tables.doc

Title of data:

- Supplementary Table 1S. Number of men 50-69 years with and with no prostate cancer by method of detection from each of the three studies
- Supplementary Table 2S. Common susceptibility loci for prostate cancer included in deriving polygenic risk score

File format: MS Word 2010

**Supplementary file:**

## Log-normal Risk Distributions

For a normally distributed risk X,

$g(y)=\frac{1}{\sigma\sqrt{2\pi}}\exp(-(y-\mu)^{2}/2\sigma^{2})$logX =Y~ N(μ,σ^2^)

i.e. the density of Y, the logarithm of the risk, is given by:

A standard result is that the mean of a log-normal distribution is given by:

E[X] = E[exp(Y)] = exp(μ+σ2/2)

E[X] = 1

Thus

μ = -σ2/2

Mean of risk score is –variance/2

And similarly

E[X^2^] = E[exp(2Y)] = exp(2μ+2σ^2^)

So that the variance of X is given by:

Var(X) = exp(2μ+2σ^2^)-exp(2μ+σ^2^)

And the relative risk to twins by

λ_M_= E[X^2^]/E[X]^2^ = exp(σ^2^)

Since risk of disease is proportional to x = e^y^, the distribution of risk (on the y scale) in cases is given by

This is also a normal distribution with parameters (μ+σ^2^,σ^2^). Thus, the risk distribution in the cases has the same shape as in the general population but shifted (on a log-scale) by σ^2^. (Hence, the mean risk in the cases is exp (μ+3σ2/2), or exp(σ^2^) times the average risk in the population).

**Supplementary Tables**

**Supplementary Table 1S.** Number of men 50-69 years with and with no prostate cancer by method of detection from each of the three studies

| Study | No. of men with screen-detected prostate cancer * | No. of men with clinically-detected prostate cancer | No. of men with no prostate cancer |
| --- | --- | --- | --- |
| ProtecT | 2,148 |  | 6,648 |
| SEARCH | 398 | 3,701 | 960 |
| UKGPCS | 867 | 2,290 |  |

* The observed prevalence of screen-detected cancers were based on ProtecT PSA-detected cases only, as the denominator population for SEARCH and UKGPCS studies are not known.

Screen-detection refers to detection as part of an organised PSA testing

**Supplementary Table 2S**. Common susceptibility loci for prostate cancer included in deriving polygenic risk score

| Locus | SNP | Proxy SNP | r2* | Effect allele | Reference allele | Effect allele frequency† | Per allele OR | 95% CI | | References | |
| --- | --- | --- | --- | --- | --- | --- | --- | --- | --- | --- | --- |
| 1q21 | rs1218582 |  |  | G | A | 0.45 | 1.06 | 1.03 | 1.09 | 1 |  |
| 1q32 | rs4245739 |  |  | C | A | 0.25 | 0.91 | 0.88 | 0.95 | 1 |  |
| 2p11 | rs10187424 | rs1009 | 0.96 | G | A | 0.41 | 0.92 | 0.89 | 0.94 | 2 |  |
| 2p11 | rs1009 | rs10187424 |  | G | A | 0.43 | 0.91 | 0.94 | 0.89 |  |  |
| 2p15 | rs721048 |  |  | A | G | 0.19 | 1.13 | 1.09 | 1.16 | 3 |  |
| 2p21 | rs1465618 |  |  | A | G | 0.22 | 1.08 | 1.05 | 1.11 | 4 |  |
| 2p24 | rs13385191 | rs13394027 | 0.89 | G | A | 0.56 | 1.15 | 1.10 | 1.21 | 5 |  |
| 2p24 | rs13394027 | rs13385191 |  | A | G | 0.22 | 1.05 | 1.01 | 1.08 |  |  |
| 2p25 | rs11902236 |  |  | A | G | 0.27 | 1.07 | 1.03 | 1.10 | 1 |  |
| 2q31 | rs12621278 |  |  | G | A | 0.05 | 0.76 | 0.69 | 0.83 | 4 |  |
| 2q37 | rs2292884 |  |  | G | A | 0.24 | 1.06 | 1.03 | 1.10 | 2,6 |  |
| 2q37 | rs3771570 |  |  | A | G | 0.15 | 1.12 | 1.08 | 1.16 | 1 |  |
| 3p11 | rs2055109 | rs1494248 | 0.78 | C | T | 0.90 | 1.20 | 1.13 | 1.29 | 7 |  |
| 3p11 | rs1494248 | rs2055109 |  | C | A | 0.30 | 0.98 | 0.96 | 1.01 |  |  |
| 3p12 | rs2660753 |  |  | A | G | 0.11 | 1.13 | 1.08 | 1.17 | 8 |  |
| 3q13 | rs7611694 |  |  | C | A | 0.41 | 0.91 | 0.88 | 0.94 | 1 |  |
| 3q21 | rs10934853 |  |  | A | C | 0.29 | 1.12 | 1.08 | 1.15 | 9 |  |
| 3q23 | rs6763931 |  |  | A | G | 0.45 | 1.03 | 1.01 | 1.06 | 2 |  |
| 3q26 | rs10936632 | rs10804839 | 0.76 | C | A | 0.48 | 0.9 | 0.88 | 0.93 | 2 |  |
| 3q26 | rs10804839 | rs10936632 |  | A | T | 0.45 | 1.09 | 1.06 | 1.12 |  |  |
| 4q13 | rs1894292 |  |  | A | G | 0.48 | 0.91 | 0.88 | 0.94 | 1 |  |
| 4q22 | rs12500426 |  |  | A | C | 0.48 | 1.09 | 1.06 | 1.12 | 4 |  |
| 4q22 | rs17021918 |  |  | A | G | 0.34 | 0.90 | 0.87 | 0.93 | 4 |  |
| 4q24 | rs7679673 |  |  | A | C | 0.39 | 0.86 | 0.83 | 0.89 | 4 |  |
| 5p12 | rs2121875 | rs1482679 | 1.00 | G | T | 0.34 | 1.05 | 1.02 | 1.08 | 2 |  |
| 5p12 | rs1482679 | rs2121875 |  | G | A | 0.33 | 1.07 | 1.04 | 1.1 |  |  |
| 5p15 | rs12653946 |  |  | A | G | 0.43 | 1.08 | 1.05 | 1.11 | 5 |  |
| 5q35 | rs6869841 |  |  | A | G | 0.21 | 1.07 | 1.04 | 1.11 | 1 |  |
| 6p21 | rs130067 |  |  | C | A | 0.21 | 1.07 | 1.03 | 1.10 | 2 |  |
| 6p21 | rs1983891 | rs913074 | 1.00 | T | C | 0.41 | 1.15 | 1.09 | 1.21 | 5 |  |
| 6p21 | rs913074 | rs1983891 |  | G | A | 0.28 | 1.07 | 1.04 | 1.10 |  |  |
| 6p21 | rs2273669 |  |  | G | A | 0.15 | 1.07 | 1.03 | 1.11 | 1 |  |
| 6q25 | rs1933488 |  |  | G | A | 0.41 | 0.89 | 0.86 | 0.92 | 1 |  |
| 6q25 | rs9364554 |  |  | A | G | 0.30 | 1.10 | 1.07 | 1.13 | 8 |  |
| 7p15 | rs10486567 |  |  | A | G | 0.22 | 0.87 | 0.83 | 0.9 | 10 |  |
| 7p21 | rs12155172 |  |  | A | G | 0.23 | 1.11 | 1.07 | 1.14 | 1 |  |
| 7q21 | rs6465657 |  |  | G | A | 0.48 | 1.12 | 1.09 | 1.15 | 8 |  |
| 8p21 | rs11135910 |  |  | A | G | 0.16 | 1.11 | 1.07 | 1.15 | 1 |  |
| 8p21 | rs1512268 |  |  | A | G | 0.45 | 1.13 | 1.10 | 1.16 | 4 |  |
| 8p21 | rs2928679 |  |  | A | G | 0.44 | 1.05 | 1.02 | 1.08 | 4 |  |
| 8q24 | rs10086908 |  |  | G | A | 0.29 | 0.87 | 0.84 | 0.90 | 11 |  |
| 8q24 | rs12543663 |  |  | C | A | 0.31 | 1.15 | 1.12 | 1.18 | 11 |  |
| 8q24 | rs1447295 |  |  | A | C | 0.13 | 1.43 | 1.39 | 1.48 | 12 |  |
| 8q24 | rs16901979 |  |  | A | C | 0.04 | 1.66 | 1.59 | 1.74 | 13 |  |
| 8q24 | rs620861 |  |  | A | G | 0.35 | 0.87 | 0.84 | 0.90 | 11 |  |
| 8q24 | rs6983267 |  |  | A | C | 0.49 | 0.8 | 0.78 | 0.83 | 14 |  |
| 9q31 | rs817826 |  |  | C | T | 0.08 | 1.41 | 1.29 | 1.54 | 15 |  |
| 9q33 | rs1571801 |  |  | A | C | 0.28 | 1.03 | 1 | 1.07 | 16 |  |
| 10q11 | rs10993994 |  |  | A | G | 0.41 | 1.23 | 1.2 | 1.26 | 8,10 |  |
| 10q26 | rs2252004 | rs2252344 | 1.00 | G | T | 0.77 | 1.16 | 1.10 | 1.22 | 7 |  |
| 10q26 | rs2252344 | rs2252004 |  | A | G | 0.10 | 0.98 | 0.94 | 1.03 |  |  |
| 10q26 | rs4962416 |  |  | G | A | 0.27 | 1.05 | 1.02 | 1.08 | 10 |  |
| 11p15 | rs7127900 |  |  | A | G | 0.21 | 1.24 | 1.21 | 1.28 | 4 |  |
| 11q13 | rs7931342 |  |  | A | C | 0.48 | 0.83 | 0.80 | 0.86 | 8,10 |  |
| 11q22 | rs11568818 |  |  | G | A | 0.44 | 0.91 | 0.88 | 0.94 | 1 |  |
| 12q13 | rs10875943 |  |  | G | A | 0.30 | 1.10 | 1.07 | 1.13 | 2 |  |
| 12q13 | rs902774 |  |  | A | G | 0.16 | 1.13 | 1.09 | 1.16 | 6 |  |
| 12q24 | rs1270884 |  |  | A | G | 0.49 | 1.07 | 1.04 | 1.10 | 1 |  |
| 14q22 | rs8008270 |  |  | A | G | 0.18 | 0.89 | 0.86 | 0.93 | 1 |  |
| 14q24 | rs7141529 |  |  | G | A | 0.49 | 1.09 | 1.06 | 1.12 | 1 |  |
| 17p13 | rs684232 |  |  | G | A | 0.36 | 1.10 | 1.07 | 1.13 | 1 |  |
| 17q12 | rs11649743 |  |  | A | G | 0.19 | 0.88 | 0.84 | 0.92 | 17 |  |
| 17q12 | rs4430796 | rs11651755 | 1.00 | A | G | 0.49 | 1.22 | 1.15 | 1.3 | 13 |  |
| 17q12 | rs11651755 | rs4430796 |  | G | A | 0.48 | 0.81 | 0.79 | 0.83 |  |  |
| 17q21 | rs11650494 |  |  | A | G | 0.08 | 1.15 | 1.1 | 1.21 | 1 |  |
| 17q24 | rs1859962 |  |  | A | C | 0.52 | 0.84 | 0.81 | 0.87 | 13 |  |
| 18q23 | rs7241993 |  |  | A | G | 0.30 | 0.92 | 0.89 | 0.95 | 1 |  |
| 19q13 | rs103294 |  |  | A | G | 0.22 | 1.00 | 0.97 | 1.04 | 15 |  |
| 19q13 | rs11672691 |  |  | A | G | 0.26 | 0.9 | 0.87 | 0.93 | 18 |  |
| 19q13 | rs2735839 |  |  | A | G | 0.13 | 0.83 | 0.79 | 0.88 | 8 |  |
| 19q13 | rs8102476 |  |  | A | G | 0.46 | 0.93 | 0.9 | 0.96 | 9 |  |
| 20q13 | rs2427345 |  |  | A | G | 0.37 | 0.94 | 0.91 | 0.97 | 1 |  |
| Xp11 | rs5945619 |  |  | G | A | 0.40 | 1.11 | 1.09 | 1.13 | 3,8 |  |
| Xq12 | rs5919432 |  |  | G | A | 0.19 | 0.96 | 0.94 | 0.99 | 2 |  |

* Correlation between two proxy SNPs

†Derived from the genotyping of 211,155 SNPs on a custom Illumina array (iCOGS) in blood from 25,074 prostate cancer cases and 24,272 controls from the international PRACTICAL Consortium^1^

Abbreviations: A, adenine; C, cytosine; G, guanine; T, tyrosine

**References**

[1] Eeles RA, Olama AA, Benlloch S et al. Identification of 23 new prostate cancer susceptibility loci using the iCOGS custom genotyping array. Nat Genet 2013; 45(4):385-391.

[2] Kote-Jarai Z, Olama AA, Giles GG et al. Seven prostate cancer susceptibility loci identified by a multi-stage genome-wide association study. Nat Genet 2011; 43(8):785-791.

[3] Gudmundsson J, Sulem P, Rafnar T et al. Common sequence variants on 2p15 and Xp11.22 confer susceptibility to prostate cancer. Nat Genet 2008; 40(3):281-283.

[4] Eeles RA, Kote-Jarai Z, Al Olama AA et al. Identification of seven new prostate cancer susceptibility loci through a genome-wide association study. Nat Genet 2009; 41(10):1116-1121.

[5] Takata R, Akamatsu S, Kubo M et al. Genome-wide association study identifies five new susceptibility loci for prostate cancer in the Japanese population. Nat Genet 2010; 42(9):751-754.

[6] Schumacher FR, Berndt SI et al. Genome-wide association study identifies new prostate cancer susceptibility loci. Hum Mol Genet 2011; 20(19):3867-3875.

[7] Akamatsu S, Takata R, Haiman CA et al. Common variants at 11q12, 10q26 and 3p11.2 are associated with prostate cancer susceptibility in Japanese. Nat Genet 2012; 44(4):426-9, S1.

[8] Eeles RA, Kote-Jarai Z, Giles GG et al. Multiple newly identified loci associated with prostate cancer susceptibility. Nat Genet 2008; 40(3):316-321.

[9] Gudmundsson J, Sulem P, Gudbjartsson DF et al. Genome-wide association and replication studies identify four variants associated with prostate cancer susceptibility. Nat Genet 2009; 41(10):1122-1126.

[10] Thomas G, Jacobs KB, Yeager M et al. Multiple loci identified in a genome-wide association study of prostate cancer. Nat Genet 2008; 40(3):310-315.

[11] Al Olama AA, Kote-Jarai Z, Giles GG et al. Multiple loci on 8q24 associated with prostate cancer susceptibility. Nat Genet 2009; 41(10):1058-1060.

[12] Amundadottir LT, Sulem P, Gudmundsson J et al. A common variant associated with prostate cancer in European and African populations. Nat Genet 2006; 38(6):652-658.

[13] Gudmundsson J, Sulem P, Manolescu A et al. Genome-wide association study identifies a second prostate cancer susceptibility variant at 8q24. Nat Genet 2007; 39(5):631-637.

[14] Yeager M, Orr N, Hayes RB et al. Genome-wide association study of prostate cancer identifies a second risk locus at 8q24. Nat Genet 2007; 39(5):645-649.

[15] Xu J, Mo Z, Ye D et al. Genome-wide association study in Chinese men identifies two new prostate cancer risk loci at 9q31.2 and 19q13.4. Nat Genet 2012; 44(11):1231-1235.

[16] Duggan D, Zheng SL, Knowlton M et al. Two genome-wide association studies of aggressive prostate cancer implicate putative prostate tumor suppressor gene DAB2IP. J Natl Cancer Inst 2007; 99(24):1836-1844.

[17] Sun J, Zheng SL, Wiklund F et al. Evidence for two independent prostate cancer risk-associated loci in the HNF1B gene at 17q12. Nat Genet 2008; 40(10):1153-1155.

[18] Al Olama A, Kote-Jarai Z, Schumacher FR et al. A meta-analysis of genome-wide association studies to identify prostate cancer susceptibility loci associated with aggressive and non-aggressive disease. Hum Mol Genet 2013; 22(2):408-415.
